# Homozygous Resistance to Thyroid Hormone *β*: Can Combined Antithyroid Drug and Triiodothyroacetic Acid Treatment Prevent Cardiac Failure?

**DOI:** 10.1210/js.2017-00204

**Published:** 2017-08-08

**Authors:** Carla Moran, Abdelhadi M. Habeb, George J. Kahaly, Christoph Kampmann, Marina Hughes, Jan Marek, Odelia Rajanayagam, Adam Kuczynski, Faraneh Vargha-Khadem, Mofeed Morsy, Amaka C. Offiah, Ken Poole, Kate Ward, Greta Lyons, David Halsall, Lol Berman, Laura Watson, David Baguley, John Mollon, Anthony T. Moore, Graham E. Holder, Mehul Dattani, Krishna Chatterjee

**Affiliations:** 1 University of Cambridge Metabolic Research Laboratories, Wellcome Trust-MRC Institute of Metabolic Science, Addenbrooke’s Hospital, Cambridge CB2 0QQ, United Kingdom; 2 Department of Paediatrics, Prince Mohammed bin Abdulaziz Hospital for National Guard & Taibah University, Al-Madinah 41511, Kingdom of Saudi Arabia; 3 Department of Medicine I, Johannes Gutenberg University Medical Center, Mainz 55101, Germany; 4 Department of Paediatric Cardiology, Johannes Gutenberg University Medical Center, Mainz 55101, Germany; 5 Department of Cardiology, Great Ormond Street Hospital for Children, London WC1N 3JH, United Kingdom; 6 Department of Neuropsychology, Great Ormond Street Hospital for Children, London WC1N 3JH, United Kingdom; 7 Department of Paediatrics, Sohag University, Egypt; 8 Academic Unit of Child Health, University of Sheffield, Sheffield S10 2TH, United Kingdom; 9 Department of Rheumatology, Addenbrooke’s Hospital, Cambridge CB2 0QQ, United Kingdom; 10 Nutrition and Bone Health, MRC Elsie Widdowson Laboratory, Cambridge CB1 9NL, United Kingdom; 11 Department of Clinical Biochemistry, Addenbrooke’s Hospital, Cambridge CB2 0QQ, United Kingdom; 12 Department of Radiology, Addenbrooke’s Hospital, Cambridge CB2 0QQ, United Kingdom; 13 Department of Audiology, Addenbrooke’s Hospital, Cambridge CB2 0QQ, United Kingdom; 14 Department of Experimental Psychology, University of Cambridge, Cambridge CB2 3EB, United Kingdom; 15 Moorfields Eye Hospital, London EC1V 2PD, United Kingdom; 16 Department of Ophthalmology, Great Ormond Street Hospital for Children, London WC1N 3JH, United Kingdom; 17 Department of Endocrinology and Section of Genetics and Epigenetics in Health and Disease, Great Ormond Street Hospital for Children and University College London Institute of Child Health, London WC1N 3JH, United Kingdom

**Keywords:** cardiac thyrotoxicosis, homozygous *THRB* mutation, resistance to thyroid hormone

## Abstract

Resistance to thyroid hormone *β* (RTH*β*) due to homozygous *THRB* defects is exceptionally rare, with only five kindreds reported worldwide. Cardiac dysfunction, which can be life-threatening, is recognized in the disorder. Here we describe the clinical, metabolic, ophthalmic, and cardiac findings in a 9-year-old boy harboring a biallelic *THRB* mutation (R243Q), along with biochemical, physiologic, and cardiac responses to carbimazole and triiodothyroacetic acid (TRIAC) therapy. The patient exhibits recognized features (goiter, nonsuppressed thyroid-stimulating hormone levels, upper respiratory tract infections, hyperactivity, low body mass index) of heterozygous RTH*β*, with additional characteristics (dysmorphic facies, winging of scapulae) and more markedly elevated thyroid hormone levels, associated with the homozygous form of the disorder. Notably, an older sibling with similar clinical features and probable homozygous RTH*β* had died of cardiac failure at age 13 years. Features of early dilated cardiomyopathy in our patient prompted combination treatment with carbimazole and TRIAC. Careful titration of therapy limited elevation in TSH levels and associated increase in thyroid volume. Subsequently, sustained reduction in thyroid hormones with normal TSH levels was reflected in lower basal metabolic rate, gain of lean body mass, and improved growth and cardiac function. A combination of antithyroid drug and TRIAC therapy may prevent thyrotoxic cardiomyopathy and its decompensation in homozygous or even heterozygous RTH*β* in which life-threatening hyperthyroid features predominate.

## Teaser

A child with severe resistance to thyroid hormone, homozygosity for mutation in thyroid hormone receptor *β* and cardiomyopathy was treated successfully with a combination of TRIAC and carbimazole.

Thyroid hormones (THs) act via nuclear receptors, encoded by separate genes (*THRA, THRB*), with differing tissue distributions (TR*α*1: central nervous system, myocardium, skeletal muscle, gastrointestinal tract; TR*β*1: liver, kidney; TR*β*2: hypothalamus, pituitary, cochlea, retina) [1].

Resistance to thyroid hormone *β* (RTH*β*), usually due to heterozygous mutations in *THRB*, is characterized by raised TH levels, nonsuppressed thyroid-stimulating hormone (TSH), and a variable phenotype encompassing both hyperthyroid (*e.g.,* failure to thrive, raised metabolic rate, tachycardia, low bone mineral density, anxiety) and hypothyroid (*e.g.,* elevated cholesterol) features [2].

Rarely, homozygous RTH*β* cases occur. Five kindreds, with eight affected individuals, have been described to date. The first documented RTH*β* siblings with a homozygous deletion of *THRB* [3, 4] exhibited moderately raised TH levels, skeletal abnormalities (stippled epiphyses, winged scapulae, pectus carinatum), deaf-mutism, and color blindness; subsequently, patients with a homozygous, single–amino acid deletion [5] and homozygous [6] or hemizygous [7] missense mutations have been documented. The clinical phenotype of absent or homozygous mutant TR*β* is severe, with very elevated TH levels, marked tachycardia, dysmorphic features, audiovisual abnormalities, and intellectual impairment. Notably, the only death attributable to RTH occurred in a homozygous RTH*β* patient, who died of heart failure during an episode of sepsis in childhood [5, 8].

Here, we describe a 9-year-old, homozygous RTH*β* child with significantly elevated TH levels, poor linear growth, and early dilated cardiomyopathy. An older sibling with similar features died of cardiac failure. A regimen combining antithyroid drug treatment to reduce TH levels with TRIAC to limit the associated TSH rise, has markedly improved growth and controlled cardiac dysfunction in our patient.

## 1. Patient and Methods

### A. Case Description

A 9-year-old boy with goiter, tachycardia, and very elevated TH levels with nonsuppressed TSH levels was investigated.

The proband was born at term, with a low birthweight of 1.9 kg (less than the third percentile), to consanguineous Arab parents. His neonatal course was unremarkable apart from a short admission to a neonatal intensive care unit after meconium aspiration. He had failure to thrive and delayed motor milestones (*e.g.*, walking and speech) during infancy. He showed signs of early developmental difficulties, including inattention, restlessness, and impulsivity, with limited awareness of danger, which necessitated high levels of adult supervision. His vocabulary is limited, and his speech is limited to short phrases or single words. Schooling is difficult; his reading and writing skills are 2 years behind those of his peer group.

Additional symptoms include anxiety, insomnia, heat intolerance, and tremor, but appetite and stool frequency are not increased. Recurrent upper respiratory tract infections (URTIs) throughout childhood necessitated tonsillectomy (age 9 years). Goiter, noted preoperatively, prompted thyroid function tests, showing a TSH of 1.9 mU/L (reference range, 0.34 to 4.6), free thyroxine (FT4) > 100 pmol/L (reference range, 12 to 22 pmol/L), and free triiodothyronine of 46.6 pmol/L (reference range, 2.8 to 7.1 pmol/L).

### B. Methods

The methods are provided in the Supplemental Data.

## 2. Results

Clinical features (goiter, hyperactivity, failure to thrive, URTI), together with very elevated TH and normal TSH levels in the proband, suggested RTH*β*.

### A. Molecular Genetic Studies

**Figure 1.**
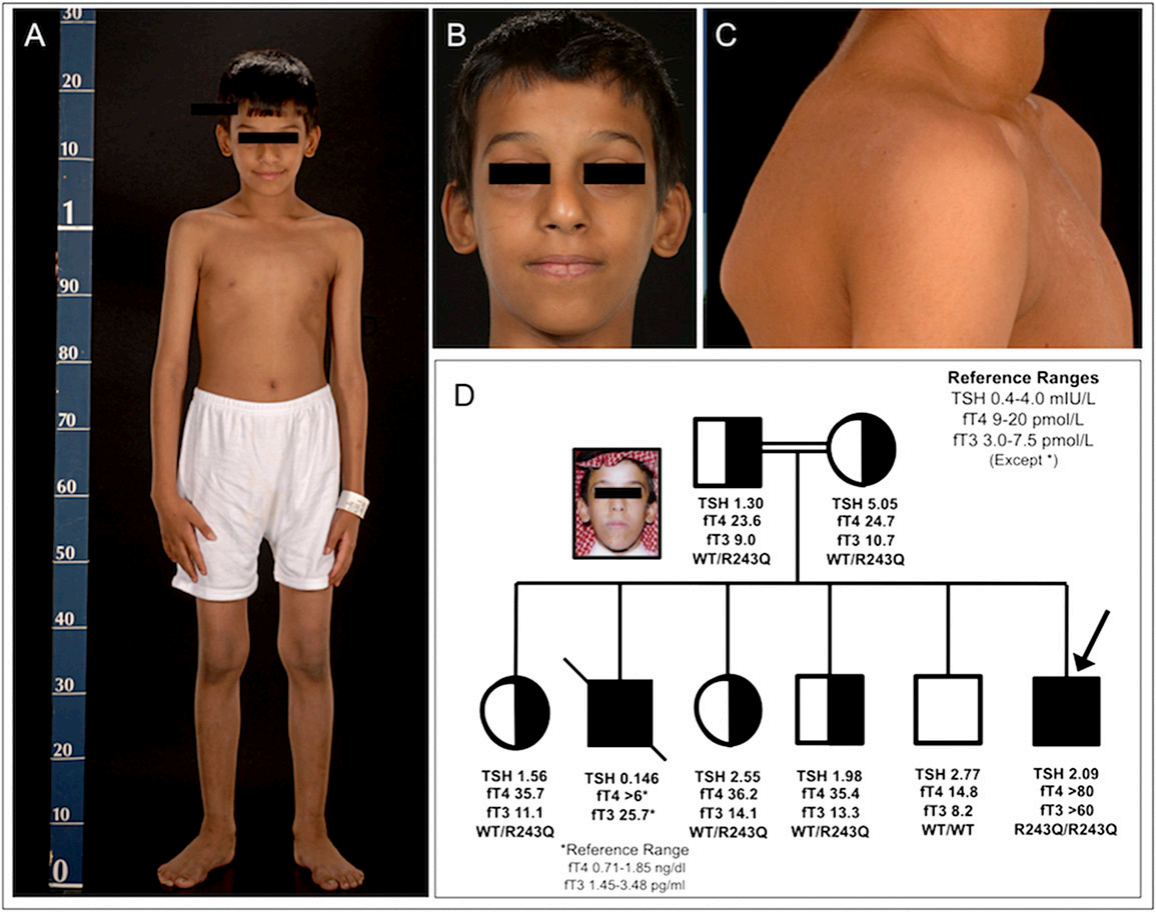
Phenotypic features of the patient and pedigree. Patient photographs illustrating thin body habitus (A), dysmorphic facial features (B), and winging of scapula and goiter (C). Pedigree (D) denoting *THRB* genotype and thyroid function tests in proband (arrow), siblings, and parents. Inset photo of deceased older brother, with similar facial features and markedly elevated thyroid hormone levels, who died of cardiac failure.

*THRB* sequencing indicated a biallelic nucleotide substitution, corresponding to homozygosity for a recognized RTH*β* mutation (arginine to glutamine change at codon 243, R243Q), in the proband. Concordant with being heterozygous for the R243Q TR*β* mutation, both parents and three siblings exhibited intermediate elevation of TH levels, and an additional further sibling with normal thyroid function showed wild-type sequence [Fig. 1(D)].

### B. Clinical Investigation

**Figure 2.**
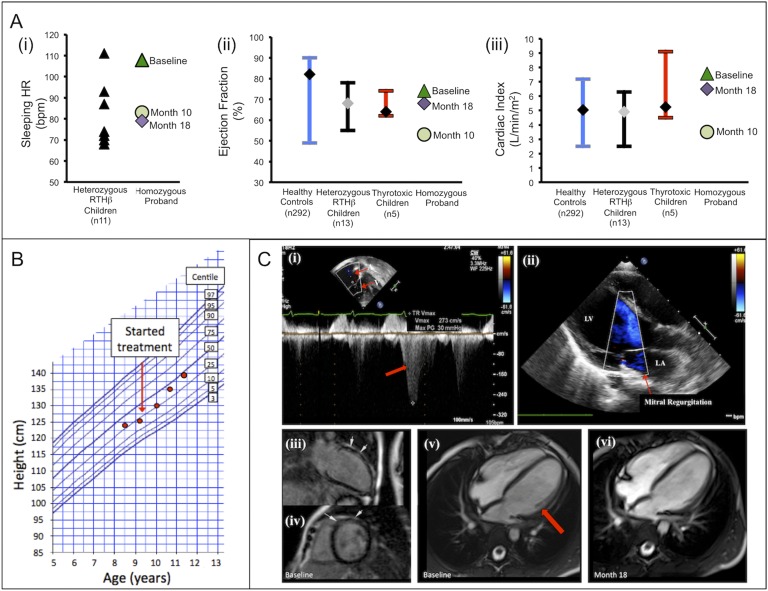
Growth and cardiac responses to treatment. (A) Heart rate (HR) and echocardiographic parameters (ejection fraction, cardiac index) in the homozygous patient at baseline, 10-month, and 18-month assessments, compared with healthy child controls, children with heterozygous RTH*β*, and children with conventional thyrotoxicosis. Bars represent the range, with diamonds representing the median. Serial height measurements (B) are plotted on an ethnically appropriate growth chart. Echocardiographic images in (Ci) and (Cii) show tricuspid and mitral regurgitation (red arrows), respectively. MRI scans show an enlarged heart with thinned, dilated LV wall (Cv) (red arrow) and areas of postcontrast myocardial enhancement (Ciii and Civ) (white arrows) at baseline assessment, with stabilization of LV dilatation and wall thinning at 18 months (Cvi).

Detailed baseline assessment of the proband was undertaken at age 9.25 years. His linear growth (height, 1.24 m; <25th percentile) [Fig. 1(A) and 2(B)] and weight (20.1 kg, <10th percentile) were subnormal. His resting energy expenditure (0.176 KJ/min per kg lean body mass) was raised, being 10% above values in age- and sex-matched healthy controls (n = 15; mean age, 10 years; 0.159 KJ/min per kg). Abnormal skeletal features included a beaked nose, flattened forehead [Fig. 1(B)], and winging of the scapulae [Fig. 1(C)] Bone age was disharmonious, being appropriate (phalanges, metacarpals) or delayed (carpal bones, distal ulna) at different sites (Supplemental Fig. 1) with normal epiphyseal development. Whole-body (Z-score, −2.2) and lumbar spine (Z-score, −2.8) areal bone mineral density by dual x-ray absorptiometry was reduced; high-resolution peripheral quantitative computed tomography showed marked reduction in trabecular bone volume at the tibia (Z-score, −4.86) and radius (Z-score, −3.59); this was very evident on three-dimensional reconstructed images (Supplemental Fig. 1). Ultrasonography confirmed an enlarged thyroid gland (volume, 19 mL; normal, 6.8 mL in 9-year-old boys [9]) of normal echotexture without nodularity. Serum thyroglobulin (79 μg/L; normal, 3 to 40 μg/L in healthy boys [10]) was significantly elevated; urinary iodine levels (125.3 μg/L; deficiency < 100 μg/L [9]) were normal.

The proband was functioning in the exceptionally low range in all neuropsychological domains tested (general intellectual ability, attention, perceptual-motor skills). At a chronologic age of 9 years, intellectual function was estimated to be equivalent to that seen at age 4 to 5 years. Reported levels of adaptive behavior were consistent with this. Brain magnetic resonance imaging (MRI) showed normal parenchyma and pituitary gland. Normal results were found for visual acuity (right, 6/6; left, 6/9), retinal imaging (fundus photography and spectral domain optical coherence tomography), and color vision. This last was assessed clinically by the Ishihara plates, the minimal color test [11], and full-field electroretinography (normal b-wave amplitude and latencies; Supplemental Fig. 2). Audiometry identified mild left-sided conductive hearing loss (Supplemental Fig. 3), likely due to a prominent jugular bulb approximating the tympanic membrane (MRI scan, data not shown).

Electrocardiography and 24-hour cardiac telemetry showed sinus tachycardia (heart rate, 125 beats/min); his sleeping heart rate (108 beats/min) was significantly elevated in comparison with that in most heterozygous RTH*β* children [Fig. 2(Ai)]. Left ventricular ejection fraction (74%) and cardiac index (7.56 L/min per m^2^) were borderline high, with a cardiac index similar to values seen in childhood thyrotoxicosis [Fig. 2(Aiii)]. His pulmonary artery pressure (39 mm Hg; normal < 25 mm Hg) was raised. Echocardiography showed mild tricuspid [Fig. 2C(i)] and mitral [Fig. 2(Cii)] regurgitation. MRI showed global cardiac enlargement, with left ventricular (LV) wall thinning [Fig. 2(Cv)]. End-systolic (37 mL/m^2^; normal, 13 to 30 mL/m^2^) and end-diastolic (84 mL/m^2^; normal, 47 to 92 mL/m^2^) LV volumes were high-normal or raised, with low-normal systolic function (LV ejection fraction, 56%; normal, 56% to 76% [12]). Postcontrast imaging showed discrete, basal anteroseptal myocardial enhancement [Fig. 2(Ciii) and 2(Civ)], suggesting focal increased tissue permeability or edema. Circulating N-terminal pro B-type natriuretic peptide (NT proBNP) concentrations (298 pg/mL; median in healthy children age 6 to 14 years, 52 pg/mL [13]) were elevated.

### C. Family History

An older brother, who died at age 13 years, had a remarkably similar medical history, with low birthweight and meconium aspiration neonatally, recurrent childhood URTIs requiring tonsillectomy, hyperactivity, and thin body habitus and facial features [Fig. 1(D)] resembling those in our patient. At age 11 years, he developed dyspnea, mitral regurgitation, and cardiac failure refractory to digoxin and diuretic therapy. His thyroid function (tested because of palpitations and sweating) was grossly abnormal [TSH, 0.14 mU/L; FT4 > 6 ng/dL (reference range, 0.71 to 1.85 ng/dL); free triiodothyronine, 25.7 pg/mL (reference range, 1.45 to 3.48 pg/mL)]. Echocardiography documented left atrial and ventricular dilatation, and he died despite diuretic and later antithyroid drug treatment.

### D. Response to Therapy

Our patient was treated with a combination of carbimazole and TRIAC over 26 months. Beta-blockade (atenolol, 50 mg daily) was also instituted but was discontinued for 72 hours before major assessments (baseline and 10, 18, and 26 months after treatment), which included serial biochemical and physiologic parameters, echocardiography, cardiac MRI, and thyroid ultrasonography.

#### D-1. Thyroid axis

**Figure 3.**
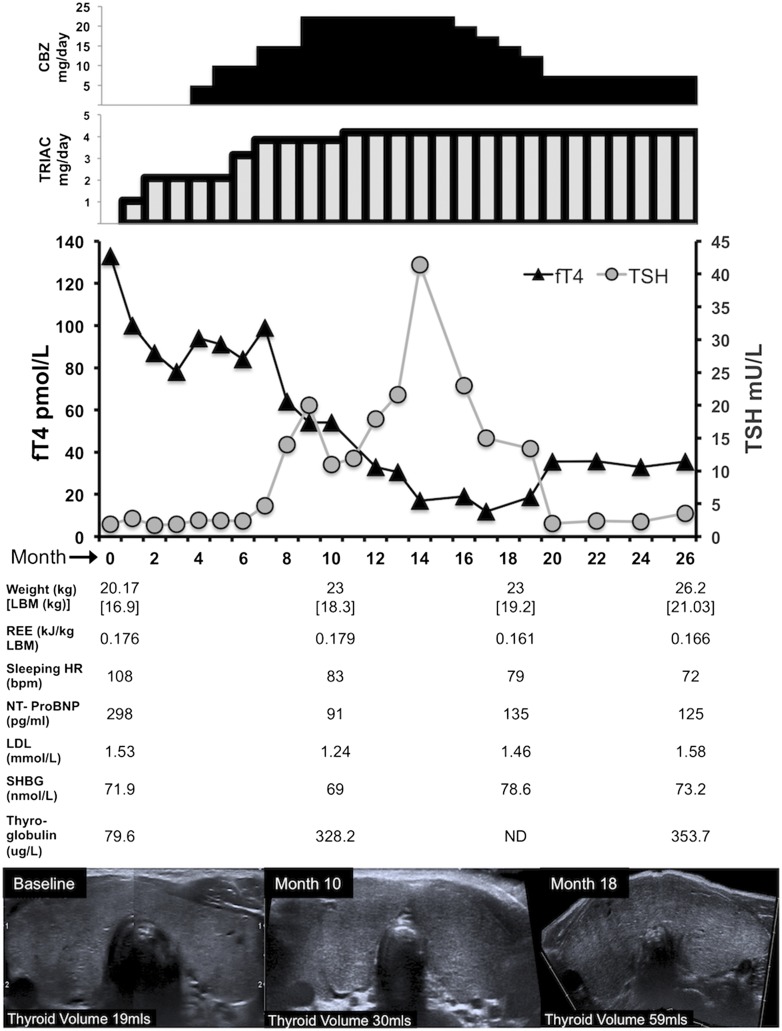
Changes in biochemical and physiologic parameters following treatment. Top bar charts denote carbimazole (CBZ) and TRIAC dosage, superimposed on a graph of serial FT4 and TSH levels over 26 months of treatment. At bottom, physiologic and biochemical parameters are tabulated at 0 (baseline) and 10, 18, and 26 months, with serial transverse thyroid ultrasonographic images and computed gland volume at similar time points. HR, heart rate; LBM, lean body mass; LDL, low-density lipoprotein; ND, not determined; REE, resting energy expenditure; SHBG, sex hormone–binding globulin.

During the first 14 months of treatment, FT4 levels fell markedly, from 133 to 14 pmol/L, with a concomitant rise in TSH, which peaked at 39 mU/L; subsequent titration of carbimazole and TRIAC achieved better, stable thyroid function (FT4, 35 pmol/L; TSH, 3.5mU/L; Fig. 3).

#### D-2. Growth and clinical response

His height velocity improved, with stature (1.39 m) crossing to between the 25th and 50th percentiles [Fig. 2(B)]. Improved concentration and diminished hyperactivity were reflected in improved school behavior and social skills.

#### D-3. Cardiac function

The patient's sleeping heart rate fell from 108 to 72 beats/min. Echocardiographic indices deteriorated at the 10-month assessment, while FT4 remained at >60 pmol/L (ejection fraction: baseline, 74% to 53% at 10 months; stroke volume index: baseline, 7.56 to 3.5 L/min per m^2^ at 10 months), but with lower FT4 levels (15 to 40 pmol/L) over the following 8 months, these parameters improved and stabilized (18 months: ejection fraction, 68%; cardiac index, 6.5 L/min per m^2^). Cardiac MRI showed an increase in LV mass (45 g at baseline to 53 g at 26 months) with absence of previous postcontrast LV wall enhancement [Fig. 2(Cvi)]. His circulating NT-proBNP levels fell from 298 to 125 pg/mL.

#### D-4. Metabolic response

Reduction in resting energy expenditure (baseline, 0.1756 to 0.166 KJ/min per kg at 26 months), was associated with substantial gain in lean body mass (16.9 kg at baseline and 21.0 kg at 26 months). Serum total cholesterol (3.1 mmol/L at baseline and 3.3 mmol/L at 26 months), low-density lipoprotein cholesterol (1.53 mmol/L at baseline and 1.58 mmol/L at 26 months), and sex hormone–binding globulin (71.9 nmol/L at baseline and 78.6 mmol/L at 26 months) have not risen substantially.

#### D-5. Goiter

The patient's thyroid volume increased (19 mL at baseline and 59 mL at 18 months) but is now stabilized (63 mL at 26 months); notably, there are no compressive features, and the texture remains nonnodular.

## 3. Discussion

We describe a patient with homozygous RTH*β*, making a total of nine individuals with this rare condition reported worldwide. Similar to previous homozygous RTH*β* cases, our patient exhibits an extreme phenotype compared with heterozygotes; the patient had FT4 6.5-fold above the upper limit of normal, as well as marked tachycardia, early cardiomyopathy and reduced trabecular bone volume. His dysmorphic facial features and winged scapulae resemble those in severe heterozygous [14] or homozygous RTH*β* patients [3, 6].

TH levels are more markedly elevated in RTH*β* patients harboring homozygous mutant TR*β* vs. deletion of *THRB* [6], suggesting that dominant negative inhibition by mutant TR*β* mediates greater resistance within the hypothalamic-pituitary-thyroid feedback axis. Computing a thyroxine (T4) resistance index (fold elevation of FT4 multiplied by TSH) in patients quantifies the degree of axis resistance [6]; compared with other homozygous RTH*β* cases (T4 resistance index of 4900 to 239,120), this patient’s T4 resistance index (836) is unusually low. TR*β*2 is also important for normal audiovisual development [15, 16]; the first homozygous *THRB* null cases exhibited profound sensorineural deafness and were color blind [3]. Hearing loss was noted in other homozygous cases [6].

Similar to TR*β*2 null mice, reduced retinal L- and M- and increased S-cone function has been documented in an RTH*β* patient with compound heterozygous TR*β* mutations [17]. Monochromacy with abnormal photopic electroretinogram (ERG) was recorded in a homozygous *THRB* null case [18]. Normal clinical testing and full-field photopic ERG in our patient exclude a major abnormality in color vision. However, because of his young age, we did not obtain specialized ERG aimed at evaluating specific cone subtypes [17, 19], such that mild, cone subtype–specific dysfunction cannot be discounted.

The combination of unimpaired color vision, normal hearing, and relatively low T4 resistance index in our patient raises the possibility that these target tissues are less refractory to TH action than in other homozygous RTH*β* cases. It is tempting to speculate that this might be due to unique functional properties of the R243Q mutation in the TR*β* background.

When studied in the TR*β*1 context, the R243Q mutant exhibits normal triiodothyronine binding in solution; however, when bound to DNA, triiodothyronine-dependent dissociation of mutant receptor homodimers or release of corepressor is markedly impaired [20, 21]. This suggests that the receptor DNA–binding domain can modulate function of the hormone-binding domain via allosteric mechanisms.

By analogy, it is tempting to speculate that by allosterically modulating transcriptional function of its divergent aminoterminal domain, the R243Q mutation is less deleterious in the TR*β*2 background, thus accounting for absent or milder phenotypes in TR*β*2-expressing target tissues.

This study used *in vivo* Xtreme CT (Scanco Medical, Zurich, Switzerland) to examine the bones of children with RTH*β*. This technique permits the evaluation of the crucial trabecular compartment and unlike dual x-ray absorptiometry is not influenced by bone or body size. In this case, it was apparent that RTH*β* was strongly linked with low trabecular bone mass rather than cortical bone mass; this observation is consistent with defective mineralization documented in homozygous TR*β* null mice [22].

Thyrotoxicosis reduces peripheral vascular resistance, induces tachycardia, and raises stroke volume [23]; the raised cardiac output and higher baseline cardiac index in our proband may reflect this. However, in chronic hyperthyroidism, inability to further increase heart rate, increase ejection fraction, or reduce systemic vascular resistance diminishes cardiac function [24] and other stressors (*e.g.*, sepsis); atrial fibrillation–mediated reduction in cardiac output or development of pulmonary hypertension can cause “decompensation” and cardiac failure [25]. Indeed, one homozygous RTH*β* case, harboring a *Δ*337T mutant TR*β* [5], died of cardiac failure in the context of systemic sepsis (Barry B. Bercu, personal communication). With marked similarities between the proband and his deceased older brother, we hypothesize that his sibling also had homozygous RTH*β* and developed heart failure following cardiac exposure to chronic hyperthyroxinemia. Thus, we sought to prevent similar TH-mediated cardiac toxicity in our patient. In this context, we reasoned that beta-adrenergic receptor blockade, reducing the chronotropic component but not the inotropic drive associated with cardiac hyperthyroidism, would be insufficient. Definitive treatment (*e.g.*, surgical or radioiodine thyroid ablation) of our patient was considered but has risks in this context. After surgery, ongoing TSH stimulation in RTH*β* prompts regrowth of remnant thyroid tissue. Thyroid ablation may require multiple, higher doses of radioiodine, with attendant risk for radiation thyroiditis–mediated TH release from the gland, precipitating thyroid storm and cardiac decompensation. Following thyroid ablation, pituitary hyperplasia does occur in RTH*β* [26], necessitating supraphysiologic thyroid hormone replacement to avoid this, but at the cost of restoring very elevated, preablation TH levels. Furthermore, no definitive criteria guide titration of postablative T4 replacement in RTH*β* The risk for suboptimal TH treatment in this childhood case is deleterious for growth, development, and neurocognition.

Although antithyroid drugs decrease TH levels effectively in conventional thyrotoxicosis, such reduction in RTH*β* cases causes exaggerated TSH elevation, overriding blockade of hormone biosynthesis and inducing goiter formation. To circumvent this, we treated our patient with TRIAC, a centrally acting TH analog known to be effective in the management of childhood RTH*β* [27–29]. This agent inhibits TSH secretion and lowers TH levels yet is relatively devoid of peripheral thyromimetic activity. Because of the magnitude of hyperthyroxinemia, TRIAC therapy alone failed to lower TH levels sufficiently. Addition of carbimazole progressively normalized FT4 levels, but this normalization was accompanied by a substantial TSH rise, with associated increase in thyroid volume (Fig. 3). Further titration of antithyroid drugs and TRIAC achieved moderate hyperthyroxinemia with normal TSH levels, reflected in lower metabolic rate, weight gain with higher lean mass, and improved growth. Increase in thyroid volume is a concern, and ongoing, regular sonographic surveillance is planned, both to assess whether the goiter has stabilized and to verify that the gland remains nonnodular, particularly as follicular tumors have been documented in homozygous TR*β* mutant mice [30].

Initial findings (tachycardia, raised cardiac index, preserved ejection fraction) in our patient were consistent with thyrotoxic cardiac status. However, significantly raised NT-proBNP levels reflected increased ventricular strain, and abnormal enhancement on MRI suggests localized myocardial edema (Fig. 2), which together raised the possibility of early dilated cardiomyopathy [31]. In that context, deterioration in cardiac index and ejection fraction in the patient, when assessed at 10 months after therapy [but with significantly elevated FT4 (>60 pmol/L)], suggested that cardiac decompensation might ensue. However, following further and sustained reduction in TH levels (<40 pmol/L), the patient's echocardiographic indices (cardiac index, ejection fraction) have improved significantly, with increased LV mass and resolution of abnormal enhancement on MRI. These changes indicate stabilization or reversal of cardiomyopathy with restored normal cardiac output.

If cardiac function and metabolic parameters remain well controlled on this combination treatment regimen, with prevention of hyperthyroid cardiomyopathy and its decompensation in the long term, we suggest that such therapy may be a useful alternative to thyroid ablation in other homozygous or even some heterozygous RTH*β* cases in which severe, potentially life-threatening hyperthyroid features predominate. Thyroidectomy, with subsequent T4 and TRIAC treatment, could be considered if the patient develops worsening heart failure or compressive symptoms due to goiter.
