# Supplementary material for: Homozygous Resistance to Thyroid Hormone β: Can Combined Antithyroid Drug and Triiodothyroacetic Acid Treatment Prevent Cardiac Failure?
Source: J Endocr Soc. 2017 Aug 8;1(9):1203–12. doi: 10.1210/js.2017-00204 (PMC5686666; doi:10.1210/js.2017-00204)
Supplement: Supplementary file 1 [file js-01-1203-sd1.doc]

**SUPPLEMENTAL DATA**

**METHODS**

All investigations were part of an ethically approved protocol and/or clinically indicated, being undertaken with parental consent**.**

**Biochemical measurements**

Free thyroid hormones (FT4, FT3) and TSH were measured using an enhanced chemiluminescence (Roche Eclia) immunoassay. Serum sex hormone binding globulin (SHBG), thyroglobulin and N-terminal pro B-type natriuretic peptide (NT-proBNP) were quantitated using chemiluminescent immunometric (Siemens IMMULITE 2000, Siemens) or immunoassay (Brahms, Thermo Scientific; Siemens Dimension). Since TRIAC cross-reacts with fT3 measurements (1) these results, verifying compliance with treatment, are not shown.

**Resting energy expenditure and body composition**

Resting energy expenditure (REE) was measured by indirect calorimetry using a ventilated canopy (GEM, GEM Nutrition, Daresbury, UK). Body composition and bone mineral density were measured using dual energy X-ray densitometry (DXA), (Lunar Prodigy, GE Medical Systems, Madison, WI). REE was expressed as KJ/min per kg of lean body mass, as measured by DXA.

**Cardiac parameters**

Sleeping heart rate (mean heart rate 2400 to 0600) was computed from a 24-72 hour recording using an accelerometer device (Actiheart, CamNtech, Cambridge, UK); heart rhythm was assessed from 24 hour cardiac telemetry. Transthoracic echocardiography (GE Healthcare) recorded standard 2D grey scale images, Doppler parameters and spectral tissue Doppler imaging and these indices were compared with datasets from healthy childhood controls and children with heterozygous RTHβ or conventional thyrotoxicosis (2). Cardiac MRI with contrast was performed using a 1.5T MR scanner (Avanto and Sonata; Siemens Healthcare, Erlangen, Germany), with acquisition of retrospectively-gated, steady state free precession cine images acquired in the short axis plane, for ventricular volumetry, and free breathing phase-contrast sequences for flow data.

**Thyroid Ultrasound**

Serial thyroid ultrasound scans were undertaken, with assessment of gland volume using an ellipsoid model, as described previously (3).

**Molecular Genetic studies**

Coding exons of *THRB* were PCR amplified from genomic DNA using specific primers and analysed by Sanger sequencing as described previously (4).

**Skeletal measurements**

Auxological parameters were plotted on charts constructed from data in healthy children from the same ethnic background (5). Bone mineral density measurements were made using quantitative CT (qCT) and high resolution peripheral quantitative CT (HR pQCT, radius and tibia, X-TremeCT 1, Scanco Medical). Results were analysed as described previously and are expressed as a standard deviation score (Z score) by comparison with the mean of an age and gender-matched healthy reference population studied at the MRC Human Nutrition Unit, Cambridge (6).

**Visual Assessment**

Visual acuity was measured using a Snellen chart. Colour vision was assessed using Ishihara plates and the minimal colour test. Retinal function was tested using full-field, photopic, electroretinography (ERG) with a white light stimulus. Imaging of the retina was performed by optical coherence tomography and retinal photography.

**Audiology**

Audiometry was performed with the patient completing a play task in response to frequency-modulated tones. Sound was presented through insert earphones (ER-3A) and bone conductor (Radioear B71) using a PC driven audiometer (Interacoustics Affinity 2.0). Otoacoustic reflexes and tympanometry were also undertaken.

**Cognitive Function**

Neuropsychological assessment was performed with the support of an interpreter. This included selected subtests from the Wechsler Intelligence Scale for Children 4^th^ UK edition (7), the Wechsler Nonverbal Scale of Ability (8), Raven’s Coloured Progressive Matrices (9), the Beery-Buktenica Developmental Test of Visual-Motor Integration 6^th^ Edn (10), the Test of Everyday Attention for Children (11) and the NEPSY-II developmental neuropsychological assessment (12). Behavioural functioning was evaluated using parent report on the Adaptive Behavior Assessment System 2^nd^ Edition (13), the Behavioural Assessment System for Children, Second Edition (14), the Children’s Communication Checklist (15), and the Conners 3rd Edition questionnaire (16).

**Supplemental References**

1. Anzai R, Adachi M, Sho N, Muroya K. Long term 3,5,3’-triiododothyroacetic acid therapy in a child with hyperthyroidism caused by thyroid hormone resistance: pharmacological study and therapeutic recommendations. Thyroid 2012; 22:1069-1075.

2. Kahaly GJ, Matthews C, Mohr-Kahaly S, Richards CA, Chatterjee VKK. Cardiac involvement in thyroid hormone resistance. J Clin Endocrinol Metab 2002; 87:204-212.

3. Zimmermann MB, Hess SY, Molinari L, De Benoist B, Delange F, Braverman LE, Fujieda K, Ito Y, Jooste PL, Moosa K, Pearce EN, Pretell EA, Shishiba Y. New reference values for thyroid volume by ultrasound in iodine-sufficient schoolchildren: a World Health Organization/Nutrition for Health and Development Iodine Deficiency Study Group Report. Am J Clin Nutr 2004; 79:231-237.

4. Adams M, Matthews C, Collingwood T, Tone Y, Beck-Peccoz P, Chatterjee VKK. Genetic analysis of 29 kindreds with generalized and pituitary resistance to thyroid hormone. J Clin Invest 1994; 94:506-515.

5. Mohammad I. El Mouzan, Abdullah A. Al Salloum, Abdullah S. Al Herbish, Peter J Foster, Mansour M. Qurashi, Ahmad A. Al Omar. The 2005 Growth Charts for Saudi Children and Adolescents (No. AR-20-63). King Abdulaziz City for Science and Technology 2009, Riyadh, KSA.

6. Nishiyama KK, MacDonald HM, Moore SA, Fung T, Boyd S, McKay HA. Cortical porosity is higher in boys compared with girls at the distal radius and distal tibia during pubertal growth: an HR-pQCR study. J Bone Miner Res 2012: 27:273-282.

7. Wechsler D. Wechsler Intelligence Scale for Children - Fourth UK Edition. London: Pearson; 2004.

8. Wechsler D, Naglieri J. A. Wechsler Nonverbal Scale of Ability. San Antonio, TX: Pearson; 2006.

9. Raven J. Coloured Progressive Matrices and Crichton Vocabulary Scale.  London: Pearson; 2004.

10. Beery KE, Beery NA, Buktenica NA. Beery-Buktenica Developmental Test of Visual-Motor Integration, Sixth Edition. Bloomington, MN: Pearson; 2010.

11. Manly T, Robertson IH, Anderson V, Nimmo-Smith I. Test of Everyday Attention for Children. London: Pearson; 1998.

12. Korkman M, Kirk U, Kemp S. NEPSY - Second Edition. San Antonio, TX: Pearson; 2007.

13. Harrison P, Oakland T. Adaptive Behavior Assessment System - Second Edition. San Antonio, TX: Pearson; 2003.

14. Reynolds CR, Kamphaus RW. Behavior Assessment System for Children, Second Edition. Minneapolis, MN: Pearson; 2004.

15. Bishop DVM. Children's Communication Checklist – Second Edition. London: Pearson; 2003.

16. Conners CK. Conners 3^rd^ Edition. Toronto: MHS; 2008.

17. Holder GE, Robson AG. Paediatric Electrophysiology: a practical approach. In: Lorenz B, Moore AT, eds. Paediatric Ophthalmology and Neuro-ophthalmology, Essentials in Ophthalmology, Vol 7, Springer, Berlin, 2005, 133-155.

**Supplementary Figure 1: Bone Age determination and high resolution peripheral quantitative CT scan (HR pQCT) in patient.**

A skeletal radiograph (Panel A), showing variably delayed bone age (carpal bones 8 yrs, distal ulna 5 yrs, phalanges 9yrs) in the patient. Three dimensional reconstruction and cutaway of the distal tibia from proband and a healthy male control of similar age (Panel B), showing marked reduction in trabecular bone density, with quantitation of this at tibia and radius below.

**Supplementary Figure 2: Electroretinography in the patient and normal subject.**

Full field electroretinography assesses the global function of the retina. The columns refer to the stimulus strength in cd.s/m^2^ and the adaptive state of the eye (DA - dark adapted, rod system dominated; LA - light adapted, cone system dominated) and are to single flashes of white light, other than the LA 30Hz recording, which shows the response to a rapidly flashing stimulus. These four responses, based upon the minimum recommended by the International Society for Clinical Electrophysiology of Vision, were recorded with peri-orbital electrodes as previously described (17). In general terms, the a-wave reflects predominantly photoreceptor function, the b-wave arising at an inner retinal level, predominantly in the retinal bipolar cells. For comparison, electrophysiological responses from a normal subject (N) are shown in the bottom row. RE; right eye, LE; left eye.

**Supplementary Figure 3: Audiogram**

Bone (red brackets) and air conduction (red circles) are normal on the right side, but there is a difference in thresholds between air and bone conduction on the left side (air conduction shown by blue crosses, bone conduction by blue brackets), signifying mild conductive hearing loss on this side.
