# Supplementary figures and images for: Homozygous Resistance to Thyroid Hormone β: Can Combined Antithyroid Drug and Triiodothyroacetic Acid Treatment Prevent Cardiac Failure?
Source: J Endocr Soc. 2017 Aug 8;1(9):1203–12. doi: 10.1210/js.2017-00204 (PMC5686666; doi:10.1210/js.2017-00204)

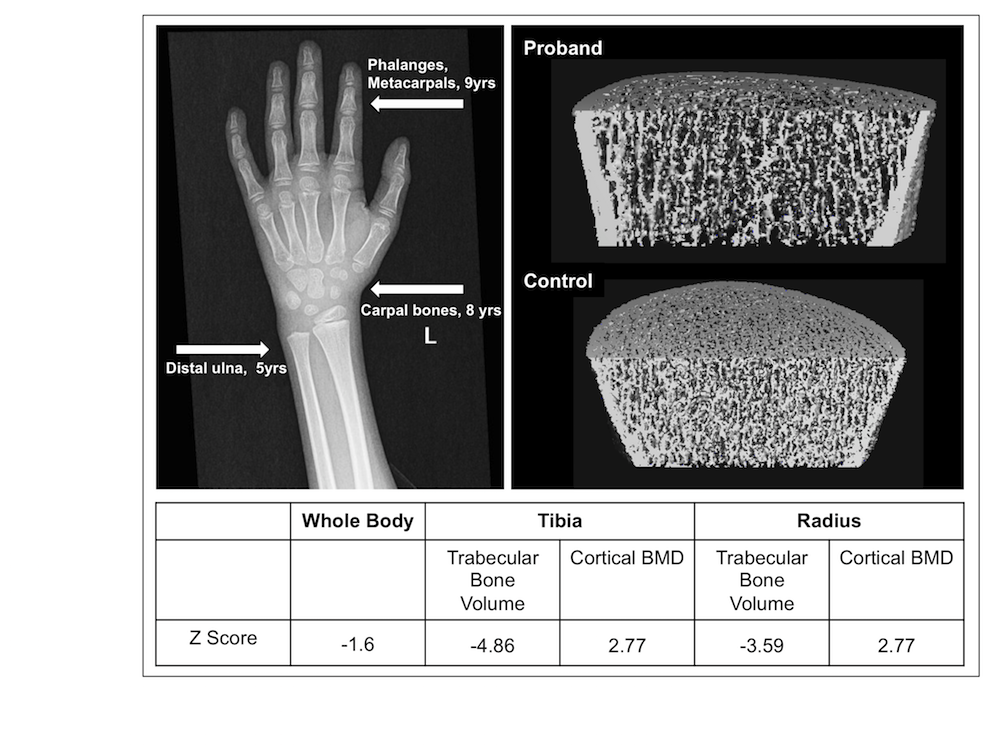

Supplement: Supplementary file 2 [file js-01-1203-sf1.tiff]

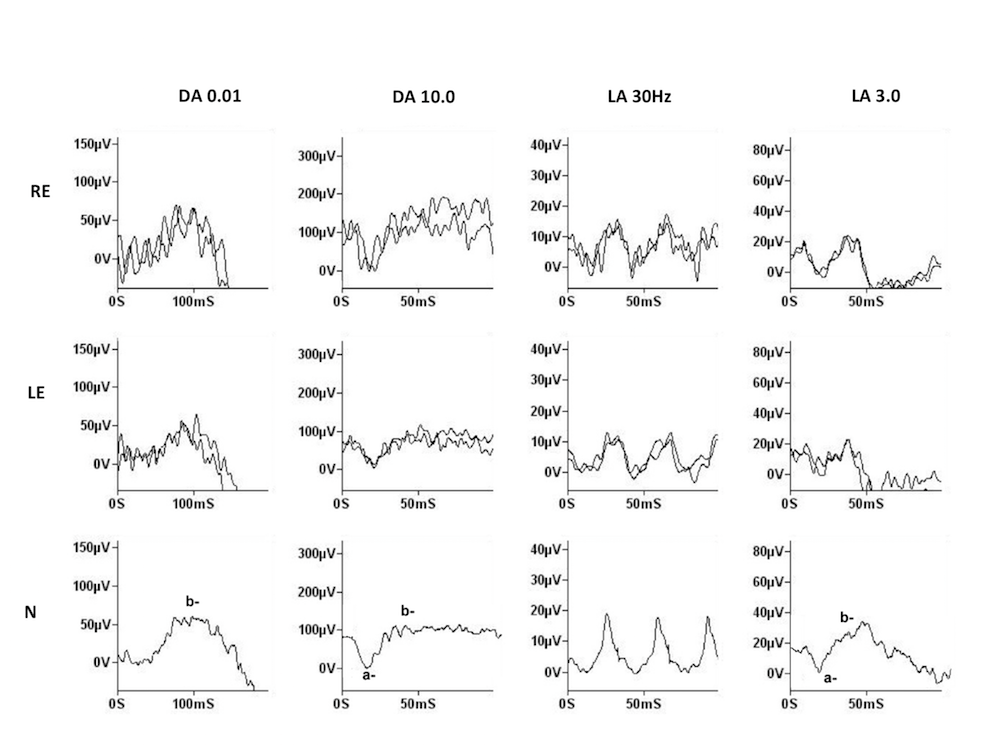

Supplement: Supplementary file 3 [file js-01-1203-sf2.tiff]

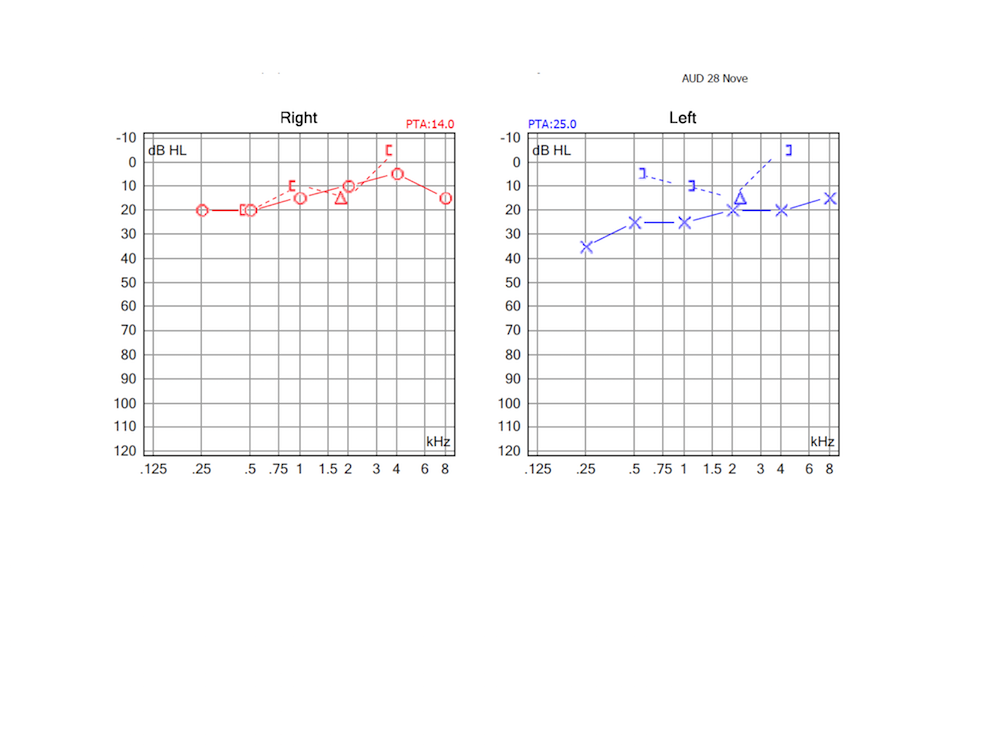

Supplement: Supplementary file 4 [file js-01-1203-sf3.tiff]
